# P-1188. Incidence and Risk Factors for Infections in Patients with B-cell Lymphoma Treated with Bispecific Antibodies

**DOI:** 10.1093/ofid/ofaf695.1381

**Published:** 2026-01-11

**Authors:** Dong Hyun Kim, Youngil Koh, Hyeon Jae Jo, Chang Kyung Kang, Pyoeng Gyun Choe, Wan Beom Park, Chan Mi Lee, Ja Min Byun, Nam Joong Kim

**Affiliations:** Seoul National University College of Medicine, Jongro-gu, Seoul-t'ukpyolsi, Republic of Korea; Seoul National University College of Medicine, Jongro-gu, Seoul-t'ukpyolsi, Republic of Korea; Seoul National University College of Medicine, Jongro-gu, Seoul-t'ukpyolsi, Republic of Korea; Seoul National University College of Medicine, Jongro-gu, Seoul-t'ukpyolsi, Republic of Korea; Seoul National University College of Medicine, Jongro-gu, Seoul-t'ukpyolsi, Republic of Korea; Seoul National University College of Medicine, Jongro-gu, Seoul-t'ukpyolsi, Republic of Korea; Seoul National University College of Medicine, Jongro-gu, Seoul-t'ukpyolsi, Republic of Korea; Seoul National University College of Medicine, Jongro-gu, Seoul-t'ukpyolsi, Republic of Korea; Seoul National University College of Medicine, Jongro-gu, Seoul-t'ukpyolsi, Republic of Korea

## Abstract

**Background:**

Bispecific antibodies (BsAbs) targeting CD3×CD20 have emerged as a promising immunotherapeutic approach in B-cell lymphoma. However, BsAbs have also led to concerns regarding infectious complications. Given the growing use of BsAbs in lymphoma, we aimed to investigate the incidence and risk factors for infections in patients with B-cell lymphoma receiving BsAb therapy.

**Figure:**
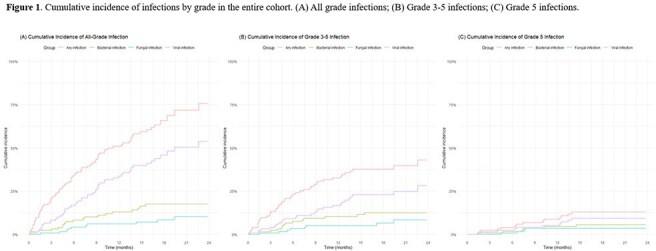

**Figure:**
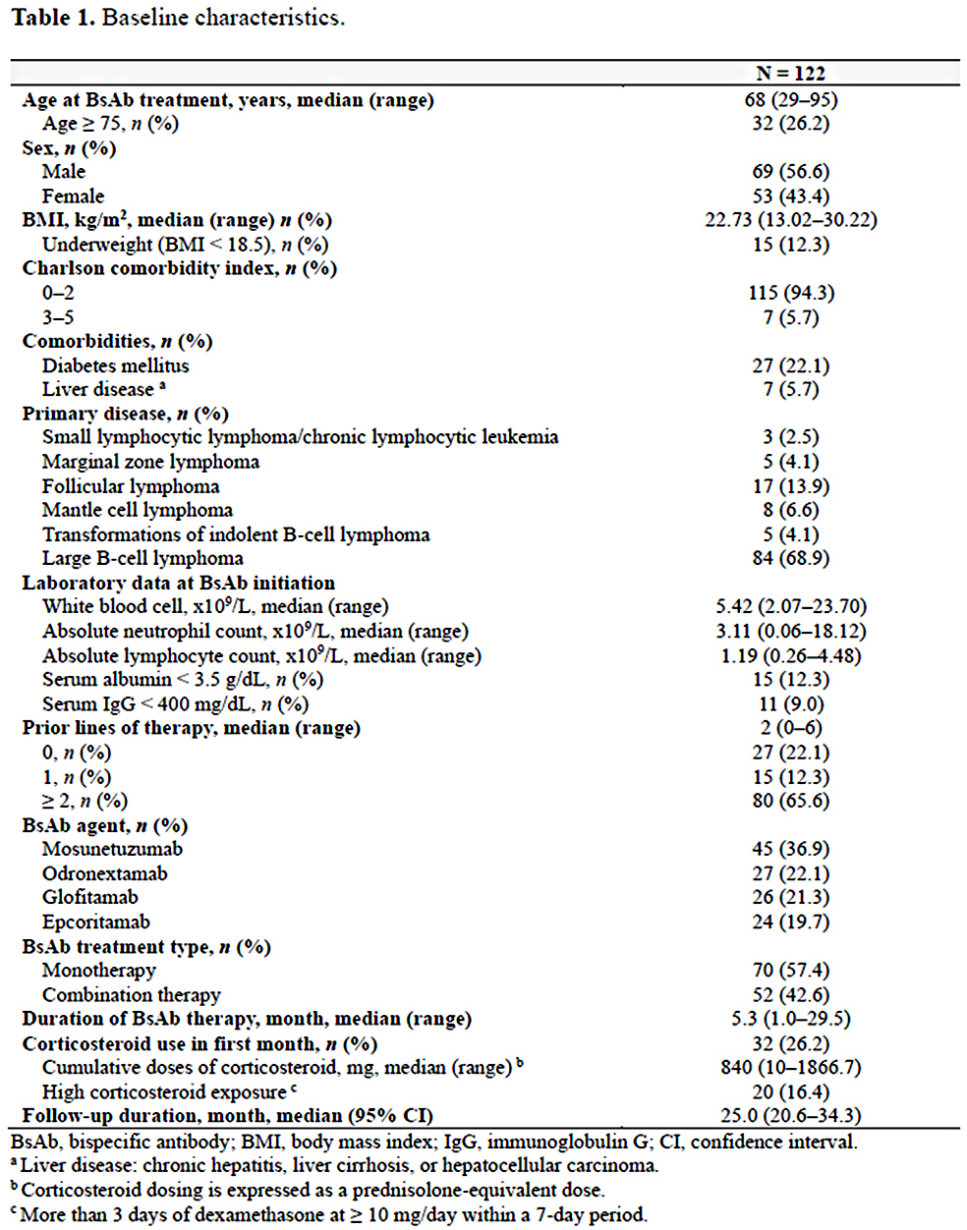

**Methods:**

This single-center, longitudinal cohort study included B-cell lymphoma patients who received CD3×CD20 BsAb therapy between 2016 and 2024 at Seoul National University Hospital. Patients who received BsAb for less than 28 days were excluded. Clinical characteristics, including comorbidities, type of lymphoma, outcomes of BsAb therapy, and episodes of infection, were collected. A multivariate Fine and Gray model was performed to identify risk factors for infectious events.

**Figure:**
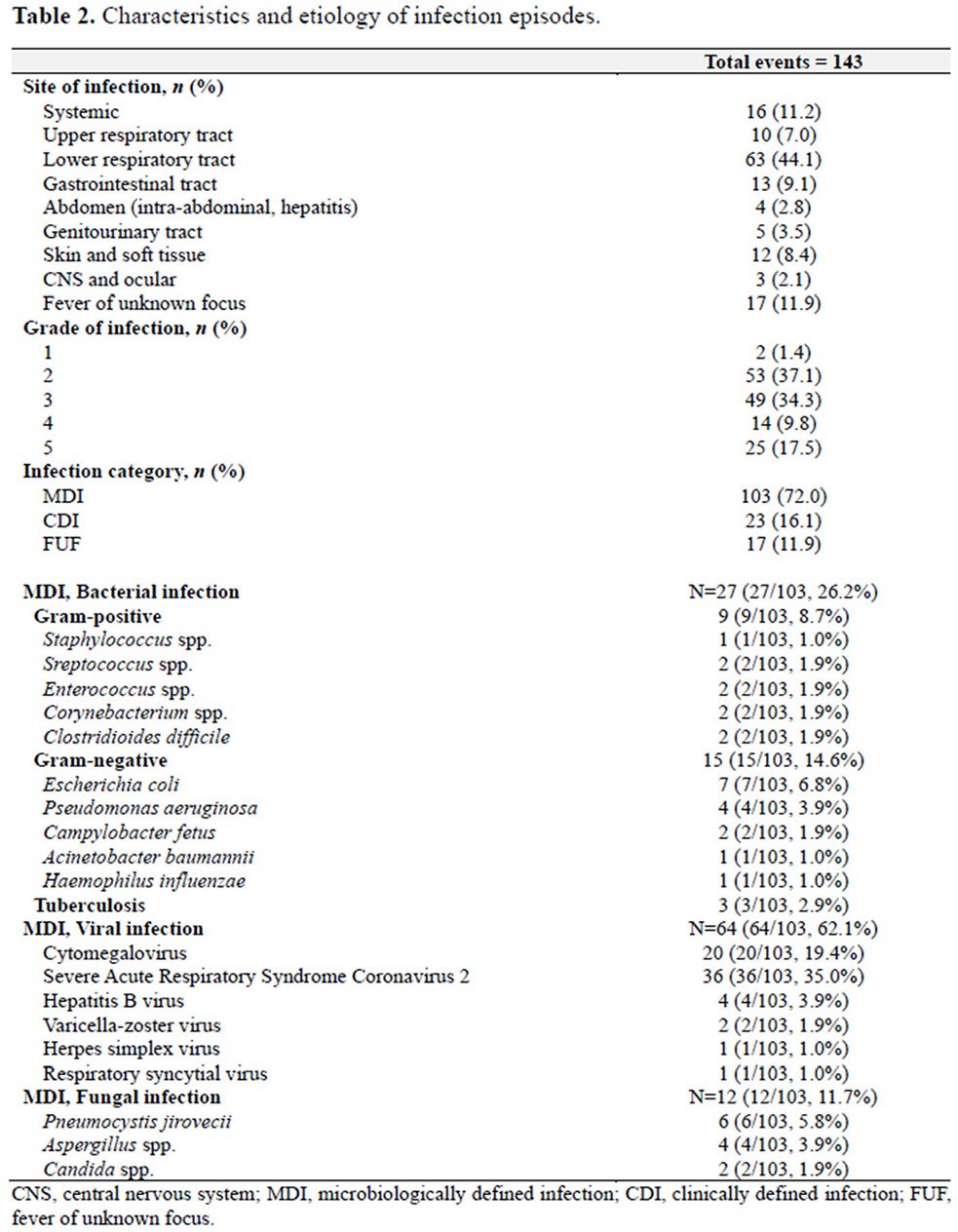

**Figure:**
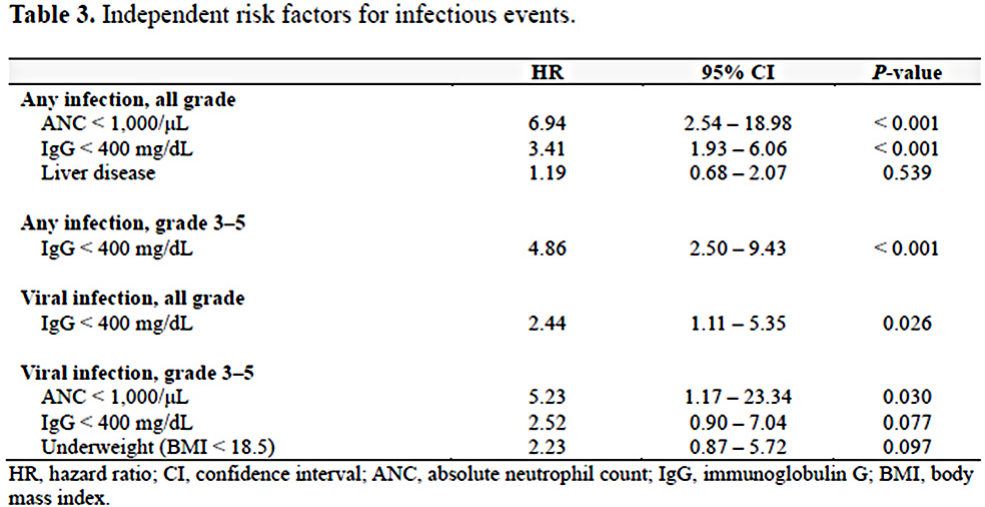

**Results:**

A total of 143 infection episodes were identified, of which 61.5% were grade ≥3. The most common sites of infection were lower respiratory tract (44.1%), followed by systemic infection (11.2%). The median time from BsAb initiation to the onset of all-grade infections was 6.5 months. Of the 122 patients, 74 (60.7%) experienced at least one infection episode, with 45 (36.9%) having grade ≥3 infections. The cumulative incidence of all-grade any infection and viral infection steadily increased over 24 months, reaching 75.6% (95% confidence interval [CI], 61.4–85.1) and 53.9% (95% CI, 41.1–65.1) at 24 months, respectively. Neutropenia (hazard ratio [HR], 6.94; 95% CI, 2.54–18.98) and hypogammaglobulinemia (HR, 3.41; 95% CI, 1.93–6.06) were associated with an increased risk of all-grade infection, while hypogammaglobulinemia was the sole independent risk factor for grade 3–5 infection (HR, 4.86; 95% CI, 2.50–9.43).

**Conclusion:**

Infections are common and clinically significant in patients with B-cell lymphoma receiving BsAb therapy, and the risk persists throughout the treatment course. To effectively mitigate infection risk, prophylactic measures against neutropenia and hypogammaglobulinemia should be implemented.

**Disclosures:**

All Authors: No reported disclosures

